# Parent-Adolescent Communication and Adolescent Delinquency: Unraveling Within-Family Processes from Between-Family Differences

**DOI:** 10.1007/s10964-019-01043-w

**Published:** 2019-06-03

**Authors:** Sabina Kapetanovic, Savannah Boele, Therése Skoog

**Affiliations:** 1 Jönköping University, Jönköping, Sweden; 2 University West, Trollhättan, Sweden; 3 Tilburg University, Tilburg, The Netherlands; 4 University of Gothenburg, Gothenburg, Sweden

**Keywords:** Parental monitoring, Delinquency, Adolescence, Parent-child relationship, Within-family, Longitudinal

## Abstract

Understanding the factors that predict adolescent delinquency is a key topic in parenting research. An open question is whether prior results indicating relative differences between families reflect the dynamic processes occurring within families. Therefore, this study investigated concurrent and lagged associations among parental behavioral control, parental solicitation, adolescent disclosure, and adolescent delinquency by separating between-family and within-family effects in three-wave annual data (*N* = 1515; *M*age = 13.01 years at T1; 50.6% girls). At the within-family level, parental behavioral control negatively predicted adolescent delinquency. Adolescent disclosure and delinquency, and adolescent disclosure and parental solicitation, reciprocally predicted each other. Parental solicitation negatively predicted parental behavioral control. The findings indicate a prominent role of adolescent disclosure in within-family processes concerning parental-adolescent communication and adolescent delinquency.

## Introduction

Researchers have performed hundreds of studies to answer the essential question of what parents can do to prevent their adolescent children from engaging in delinquent activities, including theft, vandalism, and interpersonal violence (for major reviews see Hoeve et al. 2009; Racz and McMahon 2011). Given the steep rise in delinquency in mid-adolescence (Moffitt 1993; Odgers et al. 2008), early adolescence is a critical period for taking effective measures to prevent or reduce delinquency. Although many lessons have been learned over the years, much remains unknown. For example, to what extent are aspects of parent-adolescent communication (i.e., parental control, parental solicitation, and adolescent disclosure) and adolescent delinquency reciprocally related within families over the course of early adolescence? The key problem that this study addresses is the assumption of unidirectional associations from parent-driven communication efforts (i.e., parental monitoring) to adolescent delinquency. The study puts emphasis on adolescents as actors in parent-adolescent interactions and adolescent development by addressing the reciprocity between adolescent disclosure (thus adolescent-driven communication effort), parental monitoring, and adolescent delinquency. Although studies measuring reciprocal relations between parent-driven and adolescent-driven communication efforts and delinquency are not rare (e.g., Gault-Sherman 2012; Keijsers et al. 2010; Kerr et al. 2010), to date, only one prior study has tested reciprocal associations between parent-adolescent communication efforts and adolescent delinquency both at the between- and within-family level (Keijsers 2016). Keijsers’ findings highlight that results of traditional analyses in which families are compared to each other (i.e., between-family level), do not necessarily translate to the dynamic processes happening within families (i.e., within-family level) Hamaker et al. (2015). The aim of this study is to build on and extend the vast literature on the role of parents’ and adolescents’ communication efforts in adolescent delinquency. Therefore, the reciprocal associations were examined between adolescent disclosure, parental solicitation and behavioral control, and adolescent delinquency, by separating between-family from within-family effects in a three-wave longitudinal study starting in early adolescence.

### Parents’ Efforts to Prevent Adolescent Delinquency

Throughout history, parents have been depicted as the key figures in children’s development, who shape their adolescent children’s developmental outcomes. Early control theories (e.g., Hirschi, 1969) have suggested that the basic training of children begins at home, with parents teaching their children acceptable behavior through certain parenting practices. One parenting practice that has been given a lot of attention over the past decades is parental monitoring. Parental monitoring is defined as “a set of correlated parenting behaviors involving attention to and tracking of the child’s whereabouts, activities, and adaptations” (Dishion and McMahon 1998, p. 61), and is assumed to prevent adolescents’ involvement in delinquency (Barnes et al. 2006; Hoeve et al. 2009). The idea is that parents can obtain knowledge of what their adolescents do, whom they are with, and where they are, by monitoring their adolescents’ doings and whereabouts, or by gathering of information by asking questions (i.e., parental solicitation). Another means for parents to acquire information about adolescents’ whereabouts is through regulating their adolescents’ behavior (i.e., parental behavioral control), for example by rule setting. Hence, parents can be knowledgeable about their adolescents’ whereabouts through several strategies. When parents have this information, they might potentially protect them from engaging in delinquency.

Although some studies suggest that parental socialization has negligible effect on adolescent behavioral development when adolescent genetic effects are taken account (e.g., Beaver et al. 2009; Wright et al. 2008), the vast empirical research has demonstrated a negative link between parental monitoring behaviors and adolescent delinquency, indicating that adolescents of parents who show higher levels of monitoring engage in less delinquent behavior than adolescents from parents who show lower levels of monitoring (Barnes et al. 2006; Fosco et al. 2012; Hoeve et al. 2009; Janssen et al. 2017). Hence, adolescents might be steered away from and have fewer opportunities to engage in delinquency when parents solicit information or have rules about how adolescents spend their time. Researchers therefore have advised parents to impose certain parenting strategies, including actively asking questions about their adolescents’ activities (Dishion et al. 2003; Giannotta et al. 2013; Waizenhofer et al. 2004), to maintain knowledgeable of their adolescents’ whereabouts and thus prevent adolescents from engaging in delinquency. From this perspective, processes between parental solicitation, behavioral control, and adolescent delinquency are conceptualized as parent-driven processes, in which parents’ active strategies prevent adolescents from engaging in delinquency.

### The Adolescent as the Active Mechanism

An alternative way to understand the links between parent-adolescent communication and adolescent delinquency is as a youth-driven process. In 2000, Stattin and Kerr (see also Kerr and Stattin 2000) reconceptualized the concept of parental monitoring, such that they shifted the focus to the role of adolescents’ voluntary sharing of information on their everyday activities (i.e., adolescent disclosure). They showed that adolescents’ voluntary disclosure about their everyday activities, rather than their parents’ active monitoring efforts, is linked to parental knowledge and seems to negatively predict adolescent delinquency. Consistent with Hirschi’s (1969) idea of attachment and commitment to social controls as explanation of delinquency, trusting (Kerr et al. 1999) and emotionally close (Kapetanovic et al. 2019a) relationships between parents and adolescents can provide a safe environment for adolescents to voluntarily communicate with their parents about their everyday activities. When adolescents share information about their whereabouts, parents are given opportunities to provide guidance and support, which could decrease adolescent delinquency. Indeed, some studies found that more disclosing adolescents show less delinquency than adolescents who disclose less to their parents, both concurrently (Kapetanovic et al. 2017) and over time (Keijsers et al. 2010; Smetana et al. 2006; Tilton-Weaver 2014). Thus, as a result of the reconceptualization of the concept (Stattin and Kerr 2000), parental monitoring and adolescent disclosure can be seen as mechanisms in a transactional process of interaction. A transactional model of development emphasizes the constant transformation and interdependency between persons and their environment (Loulis and Kuczynski 1997; Sameroff 2010), suggesting that adolescents are not only affected by their parents or that adolescents not only affect their parents, but that it is a constant cycle of influence between adolescents and parents.

Previous studies have investigated reciprocal associations by using a standard cross-lagged panel model, focusing on relative differences between families. Specifically, several of these studies have investigated reciprocal effects between parental knowledge and its sources (adolescent disclosure, parental behavioral control, and parental solicitation) and adolescent delinquency. For example, in a study of a sample of North American adolescents, it has been found that parental behavioral control was reciprocally linked to adolescent delinquency (Willoughby and Hamza 2011). The link between parental solicitation and adolescent delinquency has been found to be inconsistent, with studies showing either non-significant (Keijsers et al. 2010) or positive links over time (Kerr et al. 2010; Willoughby and Hamza 2011). Moreover, studies with samples of European adolescents have found that adolescent disclosure and adolescent delinquency reciprocally predicted each other (Keijsers et al. 2010; Kerr et al. 2010; Kiesner et al. 2009), such that adolescents who openly communicated with their parents, showed lower levels of engagement in delinquency one year later than adolescents who showed lower levels of voluntary disclosure. Moreover, studies indicated that links between adolescent disclosure and parental solicitation are reciprocal (Keijsers et al. 2010; Willoughby and Hamza 2011). In sum, findings of previous studies indicate that parental behavioral control, parental solicitation, adolescent disclosure, and adolescent delinquency in adolescents are reciprocally related.

### Between-Family Differences Versus Within-Family Level Processes

Despite the advances made in the literature on clarifying the potential reciprocal links between parental solicitation, behavioral control, adolescent disclosure, and adolescent delinquency, important questions about within-family processes remain unanswered. The majority of studies, including those cited above, have conducted Cross-Lagged Panel Models (CLPM) to study the reciprocal links between parent-adolescent communication efforts and adolescent delinquency. However, these models do not disentangle within-family and between-family effects (Hamaker et al. 2015; Keijsers 2016), although between-family and within-family effects have different ecological levels of inferences that might not necessarily relate to each other (Berry and Willoughby 2017; Keijsers and van Roekel 2018). In other words, the results of relative differences between families (i.e., parents who posit more rules have adolescents who engage less in delinquency than parents who posit less rules) might not translate to processes that happen within families (i.e., the adolescent decreases in delinquency after the parent posits more rules than before). Disentangling between-family from within-family effects is possible by applying Random Intercept Cross-Lagged Panel Models (RI-CLPM) (Hamaker et al. 2015), because the RI-CLPM splits between-family from within-family variance. Consequently, cross-lagged effects are estimated only at the within-family level, thus linking fluctuations within the same family over time. In this case, by applying a RI-CLPM, the question can be answered: “When parents control more or solicit more information than usual, do their adolescents engage in relatively more delinquency in the next year?”

To date, only a few studies are available that examined the longitudinal within-family processes between parent-adolescent communication efforts and adolescent delinquency are limited and inconsistent. For example, three studies have investigated the concurrent within-family and between-family links between parental behavioral control and adolescent delinquency in samples of Dutch adolescents. While one study found that increases in parental behavioral control were concurrently linked to increases in adolescent delinquency within families (Rekker et al. 2017), other studies found that increases in parental behavioral control were concurrently linked to decreases in delinquency, delinquent attitudes, and time spent in criminogenic settings (Janssen et al. 2016, 2018). To date, only one study has investigated the lagged within- and between-family effects between parental behavioral control and adolescent delinquency (Keijsers 2016), which found no significant lagged within-family processes. With respect to studies on adolescent disclosure, a negative concurrent link has been found with adolescent delinquency, indicating that increases in adolescent disclosure were related to simultaneous decreases in delinquency (Keijsers 2016; Rekker et al. 2017), but no support was found that increases in adolescent disclosure predicted later changes in adolescent delinquency within families (Keijsers 2016). Hence, existing studies that assessed within-family processes between parent-adolescent communication efforts and adolescent delinquency are limited and show inconsistent results.

Even more limited is the literature assessing within-family processes between parental monitoring and adolescent disclosure. To date, only two studies have been conducted, focusing on maternal monitoring behaviors and adolescent disclosure. The studies found that increases in maternal solicitation (Keijsers et al. 2016; Villalobos et al. 2015) were concurrently linked to increases in adolescent disclosure. No significant lagged effect was found from maternal solicitation to adolescent disclosure (Villalobos et al. 2015). In addition, increases in maternal behavioral control were concurrently linked to decreases in adolescent disclosure (Keijsers et al. 2016). Because within-family and between-family studies may provide a different picture, the reciprocal associations among parental behavioral control, parental solicitation, and adolescent disclosure need to be tested at the within-family level, which had not been done before. Thus, the transactional linkages of parents’ behaviors and adolescent disclosure, suggested by earlier empirical work at the between-person level (e.g., Keijsers and Laird 2014), are yet to be examined where they take place: at the within-family level.

## The Current Study

The aim of this study is to advance the understanding of how adolescent disclosure, parental solicitation and behavioral control, and adolescent delinquency are interrelated in early- to mid-adolescence by separating between-family differences from within-family processes. Two of the hypotheses were based on Dishion and McMahon’s (1998) idea of parental monitoring as protective of adolescent delinquency. In line with Willoughby and Hamza (2011), the first hypothesis was that parental behavioral control and adolescent delinquency would be negatively and reciprocally related, such that parental behavioral control would predict lower levels of adolescent delinquency over time, and vice versa. As earlier research shows inconsistent empirical results with respect to associations between parental solicitation and adolescent delinquency (e.g., Keijsers et al. 2010; Kerr et al. 2010), the second hypothesis was based on Dishion and McMahon’s (1998) theoretical expectation that parental solicitation would be negatively associated with adolescent delinquency. Based on Stattin and Kerr’s (2000) reconceptualization of parental monitoring and the shift of focus to adolescents as driving forces in their psychosocial development (Keijsers et al. 2010; Kerr et al. 2010), the third hypothesis was that adolescent disclosure and delinquency would be negatively and reciprocally associated over time. Given that studies suggest reciprocal associations between parental solicitation, parental behavioral control and adolescent disclosure (Willoughby and Hamza 2011), the fourth hypothesis stated that there would be positive and reciprocal associations among parental behavioral control, parental solicitation and adolescent disclosure.

## Method

### Participants

Data were used from adolescents who were part of an ongoing Swedish research program, Longitudinal Research of Development In Adolescence (LoRDIA), in which adolescents’ health, school functioning, social networks, and substance use are studied. LoRDIA was designed to follow two cohorts of adolescents in two small cities and two midsize cities in southern Sweden from the age of 12 or 13 until they are 18 years of age. Data were collected annually in schools, starting in year 2013 when the students were in 6th and 7th grade. The last data collection took place in 2018, when the latter cohort of adolescents were in 2nd grade of senior high school. Out of 2108 adolescents invited in the first wave, 318 opted out, which resulted in 1780 adolescents constituting the total sample of the study at wave 1. Out of the total sample at wave 1, 265 were absent from school on the days of the data collection, which resulted in an analytical sample of 1515 adolescents.

For this study, three waves of data from two combined cohorts of adolescents (*N* = 1515; 50.6% girls) were used, beginning in sixth grade (*n* = 781) and seventh grade (*n* = 734), respectively. The mean ages were T1: *M* = 13.01 years (SD = 0.60); T2: *M* = 14.33 years (SD = 0.64); T3: *M* = 15.65 years (*SD* = 1.09). Most students were of Swedish ethnicity (80.5%) and lived with both parents (80.6%). In terms of subjective socioeconomic status (Quon and McGrath 2014), most of the participants (62.8%) reported having as much money as their classmates, while 20.3% reported that their family had more money than their classmates’ families, and 16.8% reported that their family had less money than families of their classmates. In the analytical sample, 9.8% of students reported having a neuropsychiatric disorder such as Attention Deficit and Hyperactivity Disorder, Asperger, or Autism. To assess the representativeness of the sample use, the participants included at T1 and those who opted out were compared using available register data on demographics (gender and immigration status) and school performance (absenteeism and merit points based on grades). There were no significant differences in gender (*p* = 0.22), immigrant status (*p* = 0.07), merit points (*p* = 0.15), or absence from school (*p* = 0.60). This indicates that the sample is representative for the target sample based on gender, immigrant status, and school performance.

### Procedure

In 2013, contact was established with all primary schools in the participating municipalities and with the parents of the pupils. Students, as well as their parents, were informed about the study, its confidentiality and the voluntary basis of participation. Parents and students had the opportunity to decline consent for the students’ participation. The students replied annually to questionnaires, which were collected in the classrooms by the research team. The study received ethical approval from the Regional Research Review Board in Gothenburg, Sweden, before each data collection wave.

### Measures

#### Adolescent disclosure

This scale assessed adolescents’ voluntary disclosure to their parents about their activities during their spare time (Stattin and Kerr 2000). The scale was based on five questions, such as “If you are out at night, when you get home, do you want to tell your parents what you have done that evening?” The ratings ranged from 1 (*never*) to 3 (*often/always*) (T1: α = 0.71; T2: α = 0.71; T3: α = 0.70).

#### Parental solicitation

The scale was developed by Stattin and Kerr (2000) and measured how often parents ask about the adolescents’ unsupervised time based on five items, such as “How often do your parents initiate a conversation about things that happened during a normal day at school?” The ratings ranged from 1 (*never*) to 3 (*often/always*) (T1: α = 0.68; T2: α = 0.74; T3: α = 0.75).

#### Parental behavioral control

The scale measured ways in which parents set rules and regulations to control and regulate adolescents’ behavior. The measure, also developed by Stattin and Kerr (2000), was based on five items, such as “Do you need to have your parents’ permission to stay out late on a weekday evening?” The ratings ranged from 1 (*never*) to 3 (*often/always*) (T1: α = 0.74; T2: α = 0.81; T3: α = 0.81).

#### Adolescent delinquency

The scale, comprising seven items, measured adolescent engagement in delinquent behaviors. Six of the seven questions have been used in a nationwide school survey in Sweden (Ring 2013). Example items are “How many times have you stolen something from a shop?” and “How many times have you threatened someone to get that person’s money or other belongings?”, with a response scale ranging from 1 (*never*) to 3 (*several times*). One additional item, from Özdemir and Stattin (2011), was added to the scale “Have you beaten, kicked, or assaulted anyone at school or on the way to or from school?”, with ratings from 1 (*never*) to 3 (*once a week or several times a week*) (T1: α = 0.68; T2: α = 0.78; T3: α = 0.81).

### Attrition and Missing Values

Of those adolescents in the analytical sample (*N* = 1515), 87% remained in the study at T2 and 67% remained in the study at T3. The Missing Completely at Random test of Little (1988) was significant (χ^2^ (467) = 687, 902 *p* < .001) indicating that the assumption of missing completely random was violated. Comparisons of the baseline levels of the study variables revealed that adolescents who remained in the study at T3 were more often girls than boys (55.3% girls versus 44.7% boys, *p<* 0.001). Adolescents who remained in the study at T3 reported higher levels of adolescent disclosure (*M* = 2.53 SD = 46 versus *M* = 2.41 SD = 0.48) and parental behavioral control (*M* = 2.23 SD = 54 versus *M* = 2.17 SD = 0.55), and had lower levels of adolescent delinquency (*M* = 1.03 SD = 0.11 versus *M* = 1.06 SD = 0.17). However, the mean differences were small (Cohen 1992), ranging between 0.11 and 0.28. The most common approach to handle missing data under Missing at Random (MAR) assumption is the full information maximum likelihood (FIML) approach. Unlike listwise deletion which produces biased estimates under MAR, FIML uses all available information to produce unbiased parameter estimates and standard errors in data missing at random (Hox et al. 2017).

### Data Analysis

First, because skewness and kurtosis were unsatisfactory for delinquency at T1, T2, and T3 (skewness ranging from 5.50 at T1 to 4.24 at T3, and kurtosis ranging from 40.11 at T1 to 24.14 at T3), the full information maximum likelihood method with robust estimators (MLR) was used. This procedure can provide reliable estimates for samples with a violated assumption of normality (Rhemtulla et al. 2012).

Next, in order to test whether within-fluctuations were sufficient and thus whether within-family analyses were appropriate, the intra-class correlations (ICC) of all study variables were calculated. For adolescent disclosure, the ICC was 0.51, indicating that 51% of the variance in adolescent disclosure was explained by differences between families, and thus the remaining 49% of the variance was explained by over-time fluctuations within families. For parental solicitation, parental behavioral control, and adolescent delinquency, the ICC was 0.47, 0.36, and 0.45, respectively. Thus, the results indicated that 49–64% of variance in the variables in the study was explained by over-time fluctuations within families. Based on these results, it was concluded that within-family analyses were appropriate. Therefore, a Random Intercept Cross-Lagged Panel Model (RI-CLPM) was conducted, which distinguishes between-family variance from within-family dynamics, controls for any time-invariant confounders (such as age, race, or neuropsychiatric disorder) (Berry and Willoughby 2017; Hamaker et al. 2015), and examines how within-family fluctuations are concurrently and longitudinally related.

**Fig. 1:**
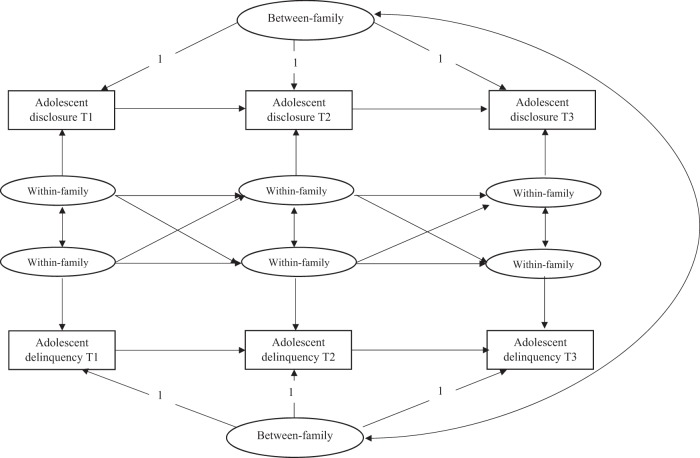
The random intercept cross-lagged panel model with two variables

The RI-CLPM was constructed with four random intercepts that represent the stable differences between families (i.e., comparing adolescents with their peers) in adolescent disclosure, parental solicitation, parental behavioral control, and adolescent delinquency. The random intercepts loaded onto the T1–T3 observed variables and each random intercept was correlated to control for the between-person correlation. Next, each observed variable was regressed on its own latent factor, with loadings set to one. Autoregressive (i.e., carry-over effects) and cross-lagged (i.e., influence of one variable on the other) within-family paths were modeled between the three time points. A two-variable random intercept model is depicted in Fig. 1. In the current study, four variables are integrated in the model. To obtain the most parsimonious model, the covariance, autoregressive stabilities, and cross-lagged paths were constrained to be the same across time points. The change in fit statistics (Satorra-Bentler scaled χ^2^—difference test, RMSEA, CFI, TLI) were tested between the unconstrained and constrained models. The model with time constraints had significantly better fit, which is why the constrained model was retained as the final RI-CLPM model (χ^2^ (*34*) = 56.456 *p* = 0.009, TLI = 0.986, CFI = 0.993, RMSEA = 0.021).

## Results

### Descriptive Statistics

Means for and correlations among the study variables (adolescent disclosure, parental solicitation, parental behavioral control, and adolescent delinquency) at each measurement wave are reported in Appendix Table 1. Overall, the correlations indicated that parental behavioral control, parental solicitation, and adolescent disclosure were negatively associated with adolescent delinquency. In addition, parental behavioral control, parental solicitation and adolescent disclosure showed positive bivariate correlations over time.

### Between-Family Level Analyses

The correlations between the random intercepts from the RI-CLPM indicated concurrent associations at the between-family level. The results suggested negative associations between the random intercepts of parental solicitation (β = −0.173) and adolescent disclosure (β = −0.256) with adolescent delinquency (see Table 2). However, the random intercepts of parental behavioral control and adolescent delinquency were not related. Furthermore, the random intercept of adolescent disclosure was positively related to the random intercepts of parental solicitation (β = 0.727) and behavioral control (β = 0.500). The random intercepts of parental solicitation and behavioral control (β = 0.694) also showed a positive association. Thus, adolescents who disclosed more and adolescents of parents who solicited more engaged less in delinquency than adolescents who disclosed less and of parents that solicited less. Additionally, adolescents whose parents solicited and controlled more disclosed more information to their parents than adolescents whose parents solicited and controlled less. Parents who solicited more engaged in more behavioral control than parents who solicited less.

**Table 2:** Random intercepts and correlations among parental behavioral control, parental solicitation, adolescent disclosure, and delinquency

		RI-CLP estimates		
	*B*	*SE*	*β*	*p*
Between-person correlations				
Disclosure RI–solicitation RI	0.051	0.007	0.727	<0.001
Disclosure RI–control RI	0.036	0.008	0.500	<0.001
Disclosure RI–delinquency RI	−0.006	0.002	−0.256	<0.05
Solicitation RI–control RI	0.052	0.008	0.694	<0.001
Solicitation RI–delinquency RI	−0.004	0.002	−0.173	<0.05
Control RI–delinquency RI	−0.003	0.002	−0.143	ns
Within-person correlations				
Disclosure T1–solicitation T1	0.056^a^	0.004	0.367	<0.001
Disclosure T2–solicitation T2	0.056^a^	0.004	0.514	<0.001
Disclosure T3–solicitation T3	0.056^a^	0.004	0.532	<0.001
Disclosure T1–control T1	0.018^b^	0.005	0.112	<0.05
Disclosure T2–control T2	0.018^b^	0.005	0.103	<0.05
Disclosure T3–control T3	0.018^b^	0.005	0.123	<0.05
Disclosure T1–delinquency T1	−0.012^c^	0.002	−0.307	<0.001
Disclosure T2–delinquency T2	−0.012^c^	0.002	−0.245	<0.001
Disclosure T3–delinquency T3	−0.012^c^	0.002	−0.246	<0.001
Solicitation T1–control T1	0.018^d^	0.006	0.099	<0.05
Solicitation T2–control T2	0.018^d^	0.006	0.108	<0.05
Solicitation T3–control T3	0.018^d^	0.006	0.112	<0.05
Solicitation T1–delinquency T1	−0.001^e^	0.001	−0.037	ns
Solicitation T2–delinquency T2	−0.001^e^	0.001	−0.027	ns
Solicitation T3–delinquency T3	−0.001^e^	0.001	−0.030	ns
Control T1–delinquency T1	−0.006^f^	0.002	−0.139	<0.05
Control T2–delinquency T2	−0.006^f^	0.002	−0.086	<0.05
Control T3–delinquency T3	−0.006^f^	0.002	−0.093	<0.05

Superscripts indicate that parameters have been set equal over time

### Within-Family Level Analyses

As shown in Table 2, concurrent negative associations were found between parental behavioral control and adolescent delinquency (T1: β = −0.139; T2: β = −0.086; T3: β = −0.093), and between adolescent disclosure and delinquency (T1: β = 0.307; T2: β = −0.245; T3: β = −0.246), indicating decreases in parental behavioral control and adolescent disclosure were concurrently related to increases in adolescent delinquency. The associations between parental behavioral control and adolescent delinquency, and between adolescent disclosure and delinquency were strongest at T1, when adolescents were 13 years old. Parental solicitation was not concurrently related to adolescent delinquency. Moreover, adolescent disclosure showed a positive concurrent association with both parental solicitation (T1: β = 0.367; T2: β = 0.514; T3: β = 0.532) and behavioral control (T1: β = 0.112; T2: β = 0.103; T3: β = 0.123), suggesting that increases in adolescent disclosure were related to simultaneous increases in parental solicitation and behavioral control. The associations between parental solicitation and adolescent disclosure were lowest at T1, followed by stronger associations at T2 and T3. Parental solicitation and parental behavioral control were also concurrently related (T1: β = 0.099; T2: β = 0.108; T3: β = 0.112), such that increases in parental solicitation co-fluctuated with increases in parental behavioral control.

**Fig. 2:**
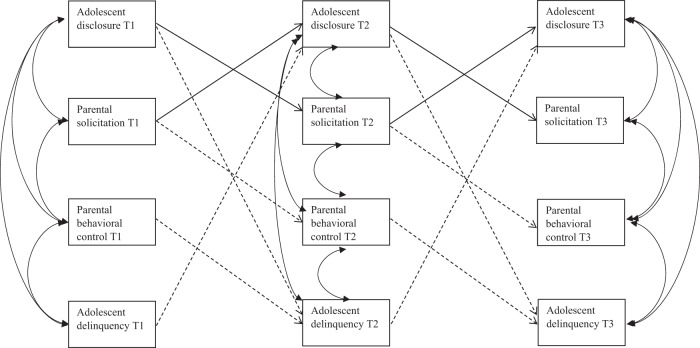
Overview of significant concurrent and lagged effects within families. Straight lines are depicting positive effects and dotted lines are depicting negative effects between the constructs

The cross-lagged effects are described in Table 3 and demonstrate that parental behavioral control predicted decreases of adolescent delinquency (T1–T2: β = −0.060; T2–T3: β = −0.064), but not the other way around. Parental solicitation did not predict adolescent delinquency over time. However, adolescent disclosure and delinquency showed a negative reciprocal association, such that an increase in adolescent disclosure predicted a decrease in delinquency the following year (T1–T2: β = −0.180; T2–T3: β = −0.155) and an increase in adolescent delinquency predicted a decrease of disclosure the following year (T1–T2: β = −0.060; T2–T3: β = −0.100). Moreover, adolescent disclosure showed a positive reciprocal association with parental solicitation, but not with control. In other words, an increase in adolescent disclosure predicted an increase in parental solicitation the following year (T1–T2: β = 0.127; T2–T3: β = 0.113) and increase in parental solicitation predicted increase in adolescent disclosure the following year (T1–T2: β = 0.173; T2–T3: β = 0.160). Furthermore, parental solicitation negatively predicted parental control (T1–T2: β = −0.102; T2–T3: β = −0.094), but parental control did not predict parental solicitation (see Fig. 2).

**Table 3:** Estimates of the cross-lagged paths between adolescent disclosure, parental solicitation, parental control, and adolescent delinquency

		RI-CLP estimates		
	*B*	*SE*	*β*	*p*
Within-person stability paths				
Disclosure T1–disclosure T2	0.188^a^	0.049	0.219	<0.001
Disclosure T2–disclosure T3	0.188^a^	0.049	0.188	<0.001
Solicitation T1–solicitation T2	0.200^b^	0.049	0.217	<0.001
Solicitation T2–solicitation T3	0.200^b^	0.049	0.208	<0.001
Control T1–control T2	0.294^c^	0.051	0.271	<0.05
Control T2–control T3	0.294^c^	0.051	0.292	<0.001
Delinquency T1–delinquency T2	0.437^d^	0.072	0.261	<0.001
Delinquency T2–delinquency T3	0.437^d^	0.072	0.435	<0.001
Within-person cross-lagged paths				
Disclosure T1–solicitation T2	0.121^e^	0.041	0.127	<0.01
Disclosure T2–solicitation T3	0.121^e^	0.041	0.113	<0.01
Disclosure T1–control T2	0.029^f^	0.059	0.022	ns
Disclosure T2–control T3	0.029^f^	0.059	0.019	ns
Disclosure T1–delinquency T2	−0.078^g^	0.018	−0.180	<0.001
Disclosure T2–delinquency T3	−0.078^g^	0.018	−0.155	<0.001
Solicitation T1–disclosure T2	0.144^h^	0.034	0.173	<0.001
Solicitation T2–disclosure T3	0.144^h^	0.034	0.160	<0.001
Solicitation T1–control T2	−0.129^i^	0.053	−0.102	<0.05
Solicitation T2–control T3	−0.129^i^	0.053	−0.094	<0.05
Solicitation T1–delinquency T2	0.004^j^	0.013	0.011	ns
Solicitation T2–delinquency T3	0.004^j^	0.013	0.010	ns
Control T1–disclosure T2	0.006^k^	0.026	0.008	ns
Control T2–disclosure T3	0.006^k^	0.026	0.009	ns
Control T1–solicitation T2	−0.031^l^	0.032	−0.039	ns
Control T2–solicitation T3	−0.031^l^	0.032	−0.044	ns
Control T1–delinquency T2	−0.021^m^	0.010	−0.060	<0.05
Control T2–delinquency T3	−0.021^m^	0.010	−0.064	<0.05
Delinquency T1–disclosure T2	−0.200^n^	0.089	−0.060	<0.05
Delinquency T2–disclosure T3	−0.200^n^	0.089	−0.100	<0.05
Delinquency T1–solicitation T2	0.108^o^	0.087	0.029	ns
Delinquency T2–solicitation T3	0.108^o^	0.087	0.050	ns
Delinquency T1–control T2	0.074^p^	0.139	0.015	ns
Delinquency T2–control T3	0.074^p^	0.139	0.024	ns

Superscripts indicate that parameters have been set equal over time

## Discussion

Many studies have assessed how aspects of parent-adolescent communication are related to adolescent delinquency (e.g., Keijsers et al. 2010; Stattin and Kerr 2000; Willoughby and Hamza 2011). However, to date, studies that unravel between-family and within-family effects are limited and inconsistent. For example, some studies found that increases in parental behavioral control are concurrently linked to increases in adolescent delinquency within families (Rekker et al. 2017), and others found the opposite (Janssen et al. 2016). Although one study revealed concurrent, but not longitudinal links, between adolescent disclosure and adolescent delinquency (Keijsers 2016), Rekker et al. (2017) found a negative, longitudinal link from disclosure to delinquency. Moreover, reciprocal processes among parental solicitation, parental behavioral control, adolescent disclosure, and adolescent delinquency have not been tested thus far. In order to provide suitable recommendations for future interventions that take place within families, within-family processes need to be disentangled from relative between-family differences. Capturing both within-family fluctuations and relative between-family differences, the current study examined how parental behavioral control, parental solicitation, adolescent disclosure, and adolescent delinquency were concurrently and longitudinally interrelated over the course of early- and mid-adolescence.

The results of the current study demonstrated somewhat different results at the between- and within-family level. At the between-family level, adolescent delinquency was negatively correlated with parental solicitation, but not with parental behavioral control. However, at the within-family level, adolescent delinquency was negatively correlated to parental behavioral control but not parental solicitation. At both levels, adolescent disclosure was negatively correlated with adolescent delinquency. Also, positive correlations emerged among adolescent disclosure, parental behavioral control, and parental solicitation. Furthermore, with respect to within-family over-time processes, the results revealed that increases in parental behavioral control predicted decreases in adolescent delinquency the following year, but not the other way around. However, fluctuations in parental solicitation did not predict later fluctuations in delinquency. Furthermore, the results showed reciprocal longitudinal links between adolescent disclosure and delinquency, and between adolescent disclosure and parental solicitation. Parental solicitation and parental behavioral control showed a unidirectional link, such that increases in solicitation predicted decreases in control. Thus, the current study provides evidence for differential processes in links among aspects of parent-adolescent communication and adolescent delinquency within families compared to between families.

### Links of Parental Behavioral Control and Parental Solicitation with Adolescent Delinquency

Based on theory (Dishion and McMahon 1998; Stattin and Kerr 2000) and empirical research (e.g., Hoeve et al. 2009), it was hypothesized that negative links between parental behavioral control and adolescent delinquency. Only one prior study tested reciprocal links at the within-family level (Keijsers 2016), finding no significant concurrent and longitudinal links between the constructs. However, in the current study, negative links were found between parental behavioral control and adolescent delinquency at the within-family level, both concurrently and over time. More specifically, adolescents reported less delinquency after their parents increased their level of strictness and demands over their adolescents’ behavior, but not the other way around. Increasing levels of parental behavioral control could be a driving force to decreases in adolescent delinquency. Although not measured in this study, increasing behavioral control may give parents more possibilities to acquire knowledge of what their adolescents are doing, as well as to protect them from getting into trouble. Hence, the protective effect of parental behavioral control on adolescent delinquency may be particularly important during periods when adolescents start spending more time in criminogenic areas (Janssen et al. 2017) or engage with deviant peers (Janssen et al. 2016). The lagged effects between parental behavioral control and adolescent delinquency were however modest in size and the findings should be interpreted with caution.

With respect to the between-family link of parental behavioral control and adolescent delinquency, the results indicated that parents who, on average, set more rules and demands on their adolescents than other parents, do not have adolescents who, on average, engage less in delinquency. One explanation for this result, which in fact contrasts the current finding on the within-family level, is that other confounding variables, such as adolescent self-control or peer delinquency as suggested by Janssen et al. (2016), mediate the associations between parental behavioral control and adolescent delinquency. Moreover, these findings are in contrast with the results from the CLPM model (see Appendix Table 4), as well as with the results from previous studies using CLPM to measure longitudinal associations between parental behavioral control and adolescent delinquency (e.g., Kerr et al. 2010). As suggested by Hamaker et al. (2015), the CLPM does not disaggregate between-family differences from within-family fluctuations, leading to a blend of different variances. Hence, the results of the RI-CLPM in the current study demonstrate the importance of disentangling within-family processes from between-family differences and show supportive evidence that increases in parental behavioral control can be followed by decreases in adolescent delinquency within families.

Reciprocal links between parental solicitation and adolescent delinquency were also examined. Parental solicitation is conceptualized as parents’ active efforts to keep track of adolescent activities by asking questions about adolescents’ whereabouts, most commonly related to parental behavioral control (Stattin and Kerr 2000; Willoughby and Hamza 2011). Although scholars have suggested that parental solicitation is unrelated to adolescent delinquency over time (Kapetanovic et al. 2019a; Keijsers et al. 2010), others indicate that it may be either protective of (Laird et al. 2010) or related to higher levels of adolescent engagement in problem behaviors over time (Kerr et al. 2010; Willoughby and Hamza 2011). Based on the theoretical implications by Dishion and McMahon (1998), a negative link was expected from parental solicitation to adolescent delinquency. However, the findings indicate that parental solicitation was related to lower levels of adolescent delinquency at the between-family level, but not at the within-family level. Thus, parents who, on average, solicited more information from their adolescents had adolescents who, on average, engaged less in delinquency than adolescents whose parents solicited less. However, increases in parental solicitation did not appear to relate to changes in adolescent delinquency within families, concurrently and over time. This finding is not conforming with the hypothesis, but it is in line with the study of Keijsers (2016). With respect to the between-family link, one explanation could be that adolescents who do not engage in delinquency do not mind their parents asking them questions about their whereabouts, because they have nothing to hide. Another explanation could be that adolescents who engage more in delinquency have less contact with their parents, for example because they spend less time at home, giving their parents less opportunities to solicit information. One explanation for the non-significant within-family link between parental solicitation and adolescent delinquency is that the effect of parental solicitation is different for each family. Thus, the families could be too heterogeneous for general principles to be defined (Keijsers and van Roekel 2018). Thus, although a negative concurrent association was found between parental solicitation and adolescent delinquency at the between-family level, no supportive evidence was found that parents’ might affect their own adolescents’ delinquent behavior by soliciting information about their whereabouts.

### Links between Adolescent Disclosure and Delinquency

Previous studies have consistently suggested that adolescents who voluntarily share information with their parents, tend to engage less in delinquency (e.g., Keijsers et al. 2010; Kerr et al. 2010). In line with previous research and the hypothesis, the results of the current study showed a negative disclosure-delinquency link at the between-family level. At the within-family level, adolescent disclosure and delinquency were reciprocally related, indicating that increases in disclosure were followed by decreases in delinquency and decreases in delinquency were followed by increases in disclosure. When adolescents share information with their parents, parents have more opportunities to give guidance and support, which in turn may result in less engagement in delinquency. However, as adolescents increase their delinquency, they could be more likely to move away from their parents and withhold information because they have something to hide. Given that the effects of adolescent disclosure on delinquency are substantively larger than effects of parental monitoring on adolescent delinquency, these findings give support to Stattin and Kerr’s (2000) reconceptualization of parental monitoring and the emphasis on adolescent agency in the parent-adolescent relationship. In contrast to the current findings, Keijsers (2016) found no significant cross-lagged links between adolescent disclosure and delinquency within families. This could be due to differences in sample size, as Keijsers (2016) had a five times smaller sample size (*n* = 309) and thus less statistical power. Nevertheless, according to the results of the current study, the reciprocal predictive links between adolescent disclosure and delinquency indicate that adolescents’ delinquent behavior is intertwined with what they tell their parents about their everyday activities.

### Can Parents Elicit Adolescent Disclosure?

As parents and adolescents mutually impact one another in their relationship (Loulis and Kuczynski 1997), it can be assumed that parents’ behavior affects adolescents’ willingness to share information. Links between parental solicitation and adolescent disclosure have been found in earlier studies (Keijsers et al. 2010; Tokić Milaković et al. 2017). Although Villalobos et al. (2015) tested concurrent within-family processes in links between parental solicitation and adolescent disclosure, the current study assessed the reciprocal links between these constructs at the within-family level. In addition, the within-family processes between parental behavioral control and adolescent disclosure were also tested. The findings at the between-family level indicate that adolescent disclosure is related to higher levels of parental solicitation and parental behavioral control. This is in line with other studies using CLP designs in which between-family and within-family variances are blurred (e.g., Kapetanovic et al. 2019b; Willoughby and Hamza 2011). Importantly, the literature is extended by providing the first evidence that parental solicitation and adolescent disclosure demonstrate a reinforcing cycle within families. Hence, increases in solicitation were related to increases in adolescent disclosure to parents and vice versa. These findings indicate that adolescent disclosure and parental solicitation are intertwined aspects of parent-child communication (Keijsers et al. 2010). When adolescents talk to their parents about their everyday activities, parents are better able to have open communication with their children, with less risk of being perceived as intrusive by adolescents. Instead, parents’ questions may be perceived by adolescents as an act of care and providing them with an opportunity to be more open about their lives (Tokić Milaković et al. 2017). If adolescents do not engage in information sharing, parents may withdraw their interest and solicit less (Keijsers and Laird 2014). Showing interest in their adolescents’ activities may be advantageous for parents because it permits them to stay involved in their adolescents’ lives.

However, one important issue is how adolescents perceive parents’ questions and queries. Although not measured in the current study, it is possible that the link between parental solicitation and adolescent disclosure depends on whether or not adolescents believe their parents have jurisdiction over the issues in adolescents’ lives (Smetana et al. 2005). If adolescents perceive their parents’ questions to be legitimate, it could be more likely that they share information. If they do not perceive parents’ questions as legitimate, adolescents could perceive their parents’ questions as intrusive (Kakihara and Tilton‐Weaver 2009) and disclose less, which in turn could have an effect on adolescent behavior. Thus, in future research it would be interesting to investigate the moderating role of legitimacy beliefs of parents’ monitoring efforts in the link between parental solicitation and adolescent disclosure.

Moreover, there were some unexpected findings in the current study. Although the positive between-family correlation between parental solicitation and parental behavioral control was in line with other studies (Kapetanovic et al. 2017; Stattin and Kerr 2000), at the within-family level, increases in parental solicitation predicted decreases in parental behavioral control. This finding is in contrast with the results from a standard cross-lagged modeling design (see Appendix Table 4, and e.g., Willoughby and Hamza 2011) that showed a positive longitudinal link between parental behavioral control and parental solicitation. In other words, the results from the CLPM indicated that controlling adolescents’ free time is followed by more solicitation, whereas when between- from within-family variances are separated another direction of effects emerges. Thus, as suggested by the findings in the current study, parents tend to decrease their behavioral control after they increase their solicitation. As discussed earlier, it is possible that parents decrease their level of control when they and their adolescents engage in reciprocal communication. As increased solicitation apparently results in increases in adolescent disclosure, parents are able to relax their rules and demands (i.e., behavioral control). Parents may do so because they feel more involved in their adolescents’ lives as a result of the increased parent-adolescent communication.

In developmental research, the interpretation of the current findings should be informed by awareness of the societal context of the developing adolescents and their parents. This study was set in Sweden. Being a family in Sweden is in some ways different compared to what it is like in other societies. Culturally, the concept of parenting in Sweden has a strong emphasis on democratic family interactions (Stattin et al. 2011). Open communication between parents and adolescents, parental control and warmth, and adolescent influence in family decisions are some of the features that characterizes Swedish families in comparison to families in other contexts. Thus, the emphasis on democratic parenting in Swedish families could influence the findings. At the same time, it should be noted that studies indicate that parents in other Western contexts, including Europe and the United States, move toward more progressive parenting attitudes (Lansford et al. 2011; Putnick et al. 2012). Past findings include samples from European societies, including Sweden (e.g., Kerr et al. 2010; Keijsers 2016; Rekker et al. 2017; Tokić Milaković et al. 2017), and North America (Villalobos et al. 2015; Willoughby and Hamza 2011). In line with the current study, past research has typically included large samples of adolescents from dual-parent households. Nevertheless, studies in contexts with other views on parenting and child rearing are needed to test the replicability of the findings. Specifically, more research is needed to address the question of the reciprocal links between parent-adolescent communication and adolescent delinquency in culturally or socially diverse sample of adolescents and families in non-Western cultures (Smetana 2017).

Apart from the study’s strengths, some limitations should be mentioned. First, data consisted solely of adolescents’ self-reports. Although this is common practice in parenting research, it could lead to several problems including response bias. On the other hand, and in line with Kuczynski’s model of parent-adolescent interactions, adolescents act on what they perceive (Kuczynski and De Mol 2015), which is why asking adolescents how they perceive their parents could be an appropriate method. Second, the within-family processes in the RI-CLMP model are averaged within-family processes, meaning that such processes might not necessarily apply to all individual families. Although heritability in within-family processes is controlled for in the current study (Berry and Willoughby 2017), using a sample of monozygotic (MZ) and dizygotic (DZ) twins could provide a more nuanced picture of family fluctuations among siblings within families. Indeed, studies have found that genetic factors are significantly accountable for individual differences in adolescent delinquency (Wright et al. 2008) and that between-family links between parenting and adolescent behavior are moderated by adolescents’ temperament (Belsky and Pluess 2009; Kapetanovic et al. 2019b). However, is has not yet been studied whether adolescent personality moderates the within-family link between parenting and adolescent behavior. Moreover, based on ecological theories (e.g., Bronfenbrenner 1986; Sameroff 2010), contextual differences between families may result in variation in the within-family processes, and empirical evidence suggest that the socio-economic status of the family explains differences between families in within-family processes of parental control and adolescent delinquency (Rekker et al. 2017). Hence, the average within-family processes should be generalized with caution (Keijsers and van Roekel 2018) and future research can offer a deeper insight concerning to what extent these processes apply to each family. Third, within-family processes were examined from year-to-year and little is known whether and how these processes operate on a shorter time scale, such as from month-to-month or day-to-day (Keijsers et al. 2016; Villalobos et al. 2015). The coercion theory, for example, assumes that hostile parent-child interactions, taking place in the moment, affect the development of antisocial behavior that evolves on a macro time scale (Granic and Patterson 2006; Patterson 1982). Hence, it might be meaningful to assess how aspects of parent-adolescent communication (e.g., solicitation and disclosure) relate to adolescent delinquency on a shorter time scale. Finally, as earlier studies suggest, parental behavioral control and solicitation can be perceived as overly controlling (Kakihara and Tilton‐Weaver 2009) or intrusive (Hawk et al. 2008). Therefore, as suggested previously in the article, future research might include assessments of adolescents’ interpretation of parental efforts, such as behavioral control or solicitation, in the models of parent-adolescent communication efforts and adolescent psychosocial outcomes.

## Conclusion

Parents’ and adolescents’ communication efforts are considered protective of adolescent involvement in delinquency over time (Kerr et al. 2010; Willoughby and Hamza 2011). Although studies acknowledge reciprocity in parent-adolescent interactions (Gault-Sherman 2012; Keijsers et al. 2010), previous research was not able to disentangle the stable differences between families from processes that happen within families over time. The current study tested reciprocal associations between parent-adolescent communication efforts (i.e., parental behavioral control, parental solicitation, and adolescent disclosure) and adolescent delinquency within families and contributes to the literature on parent-adolescent communication and adolescent delinquency in at least three critical ways. First, the longitudinal design allowed for studying the reciprocal processes in parent-adolescent interactions taking place within families. Next, the novel analytical approach, in which relative differences between families and overt-time processes within families are separated, provided a novel understanding of how parent-adolescent communication efforts and adolescent delinquency are related. The findings support that parents and adolescents reciprocally affect each other and both might contribute to adolescent delinquency (Kuczynski and De Mol 2015). Moreover, although parental behavioral control and solicitation are generally seen as parental monitoring practices (Willoughby and Hamza 2011), the findings in the current study indicate that parental solicitation is more likely to be an aspect of parent-adolescent communication, possibly contributing to a stronger parent-adolescent relationship. When adolescents and parents are responsive to one another, their relationship becomes stronger, which can result in parents relaxing their authority over the adolescents and being more supportive of adolescents’ autonomy development instead. As a result of changing interactions between parents and their adolescents, adolescents may engage less in delinquency. Acknowledging adolescents’ behaviors and their own communication efforts might help parents adjust their parenting practices to more successfully help their adolescents to stay away from harm. In terms of implications for practices, the current findings shed new light on how families differ from each other regarding parent-adolescent interactions (between-family effect) by showing in which families adolescents are at risk for engaging in more delinquency. Importantly, the findings can be used to help unravel how parent- and adolescent-driven communication efforts and adolescent delinquency operate within individual families over time (within-family effect). This is an important first step in identifying the causal processes in parent-adolescent interactions and adolescent development (Keijsers 2016). Ultimately, the understanding of the dynamic processes within families could be used to inform the design of preventive interventions for parents and families.

## Appendix

Table 1

**Table 1:** Means and correlations among adolescent disclosure, parental solicitation, parental behavioral control, and adolescent delinquency

	Adolescent disclosure T1	Parental solicitation T1	Parental behavioral control T1	Adolescent delinquency T1	Adolescent disclosure T2	Parental solicitation T2	Parental behavioral control T2	Adolescent delinquency T2	Adolescent disclosure T3	Parental solicitation T3	Parental behavioral control T4	Adolescent delinquency T3
Adolescent disclosure T1	1											
Parental solicitation T1	0.491**	1										
Parental behavioral control T1	0.262**	0.317**	1									
Adolescent delinquency T1	−0.289**	−0.083**	−0.132**	1								
Adolescent disclosure T2	0.527**	0.413**	0.203**	−0.164**	1							
Parental solicitation T2	0.350**	0.475**	0.217**	−0.081**	0.606**	1						
Parental behavioral control T2	0.166**	0.168**	0.402**	−0.056	0.220**	0.227**	1					
Adolescent delinquency T2	−0.255**	−0.114**	−0.142**	0.496**	−0.311**	−117**	−0.184**	1				
Adolescent disclosure T3	0.433**	0.337**	0.157**	−0.108**	0.587**	0.465**	0.239**	−0.188**	1			
Parental solicitation T3	0.291**	0.403**	0.194**	−0.078*	0.434**	0.568**	0.202**	−0.049	0.621**	1		
Parental behavioral control T3	0.114**	0.120**	0.312**	−0.070*	0.142**	0.112**	0.496**	−0.075*	0.175**	0.214**	1	
Adolescent delinquency T3	−0.175**	−0.096**	−0.047	0.328**	−0.256**	−0.112**	−0.132**	0.521**	−0.273**	−0.078**	−0.071*	1
Mean (SD)	2.49 (0.47)	2.16 (0.48)	2.21 (0.54)	1.04 (0.13)	2.42 (0.42)	2.18 (0.44)	2.12 (0.56)	1.06 (0.18)	2.43 (0.42)	2.20 (0.45)	1.99 (0.58)	1.06 (0.17)

**p* < 0.05 and ***p* < 0.001

Table 4

**Table 4:** Correlations and cross-lagged associations among parental behavioral control, parental solicitation, adolescent disclosure and delinquency

	CLP estimates			
	*B*	*SE*	*β*	*p*
Correlations				
Disclosure T1–solicitation T1	0.079^a^	0.003	0.394	<0.001
Disclosure T2–solicitation T2	0.079^a^	0.003	0.544	<0.001
Disclosure T3–solicitation T3	0.079^a^	0.004	0.564	<0.001
Disclosure T1–control T1	0.037^b^	0.004	0.160	<0.001
Disclosure T2–control T2	0.037^b^	0.004	0.199	<0.001
Disclosure T3–control T3	0.037^b^	0.004	0.201	<0.05
Disclosure T1–delinquency T1	−0.013^c^	0.001	−0.232	<0.001
Disclosure T2–delinquency T2	−0.013^c^	0.001	−0.231	<0.001
Disclosure T3–delinquency T3	−0.013^c^	0.001	−0.247	<0.001
Solicitation T1–control T1	0.050^d^	0.004	0.207	<0.001
Solicitation T2–control T2	0.050^d^	0.004	0.236	<0.001
Solicitation T3–control T3	0.050^d^	0.004	0.247	<0.001
Solicitation T1–delinquency T1	−0.003^e^	0.001	−0.051	<0.05
Solicitation T2–delinquency T2	−0.003^e^	0.001	−0.047	<0.05
Solicitation T3–delinquency T3	−0.003^e^	0.001	−0.052	<0.05
Control T1–delinquency T1	−0.008^f^	0.002	−0.115	<0.001
Control T2–delinquency T2	−0.008^f^	0.002	−0.095	<0.001
Control T3–delinquency T3	−0.008^f^	0.002	−0.102	<0.001
Stability paths				
Disclosure T1–disclosure T2	−0.422^g^	0.022	0.435	<0.001
Disclosure T2–disclosure T3	−0.422^g^	0.022	0.416	<0.001
Solicitation T1–solicitation T2	0.403^h^	0.023	0.397	<0.001
Solicitation T2–solicitation T3	0.403^h^	0.023	0.407	<0.001
Control T1–control T2	0.456^i^	0.022	0.419	<0.001
Control T2–control T3	0.456^i^	0.022	0.452	<0.001
Delinquency T1–delinquency T2	0.614^j^	0.055	0.438	<0.001
Delinquency T2–delinquency T3	0.614^j^	0.055	0.583	<0.001
Cross-lagged paths				
Disclosure T1–solicitation T2	0.154^k^	0.023	0.147	<0.001
Disclosure T2–solicitation T3	0.154^k^	0.023	0.144	<0.001
Disclosure T1–control T2	0.053^l^	0.033	0.040	ns
Disclosure T2–control T3	0.053^l^	0.033	0.039	ns
Disclosure T1–delinquency T2	−0.048^m^	0.011	−0.116	<0.001
Disclosure T2–delinquency T3	−0.048^m^	0.011	−0.107	<0.001
Solicitation T1–disclosure T2	0.154^n^	0.020	0.164	<0.001
Solicitation T2–disclosure T3	0.154^n^	0.020	0.164	<0.001
Solicitation T1–control T2	−0.005^o^	0.030	−0.004	ns
Solicitation T2–control T3	−0.005^o^	0.030	−0.004	ns
Solicitation T1–delinquency T2	0.000^p^	0.009	0.001	ns
Solicitation T2–delinquency T3	0.000^p^	0.009	0.001	ns
Control T1–disclosure T2	0.028^r^	0.014	0.035	ns
Control T2–disclosure T3	0.028^r^	0.014	0.037	ns
Control T1–solicitation T2	0.041^s^	0.015	0.047	<0.05
Control T2–solicitation T3	0.041^s^	0.015	0.052	<0.05
Control T1–delinquency T2	−0.012^t^	0.007	−0.034	ns
Control T2–delinquency T3	−0.012^t^	0.007	−0.036	ns
Delinquency T1–disclosure T2	−0.117^u^	0.048	−0.035	<0.05
Delinquency T2–disclosure T3	−0.117^u^	0.048	−0.049	<0.05
Delinquency T1–solicitation T2	0.086^v^	0.053	0.024	ns
Delinquency T2–solicitation T3	0.086^v^	0.053	0.034	ns
Delinquency T1–control T2	0.049^x^	0.086	0.011	ns
Delinquency T2–control T3	0.049^x^	0.086	0.015	ns
Fit indices				
χ^2^ (*df*)		191.821 (44)		
*p*		0.000		
TLI		0.931		
CFI		0.954		
RMSEA		0.047		

Equal superscripts refer to parameter constrains
